# 4-(Diphenyl­phosphan­yl)benzoic acid

**DOI:** 10.1107/S1600536811034234

**Published:** 2011-08-27

**Authors:** Pei-Hua Zhao, Fu-Yu Sun, Jun-Jie Liu

**Affiliations:** a Research Center for Engineering Technology of Polymeric Composites of Shanxi Province, School of Materials Science and Engineering, North University of China, Taiyuan 030051, People’s Republic of China

## Abstract

In the title compound, C_19_H_15_O_2_P, the dihedral angles between the benzoic acid ring and the phenyl rings are 75.64 (7) and 80.88 (7)°; the dihedral angle between the phenyl rings is 81.35 (7)°. In the crystal, inversion dimers linked by pairs of O—H⋯O hydrogen bonds generate *R*_2_^2^(8) loops between the head-to-head carb­oxy­lic acid groups.

## Related literature

For background to phosphine ligands, see: Dydio *et al.* (2011 ▶). For water-soluble phosphines, see: Katti *et al.* (1999 ▶); Pinault & Bruce (2003 ▶).
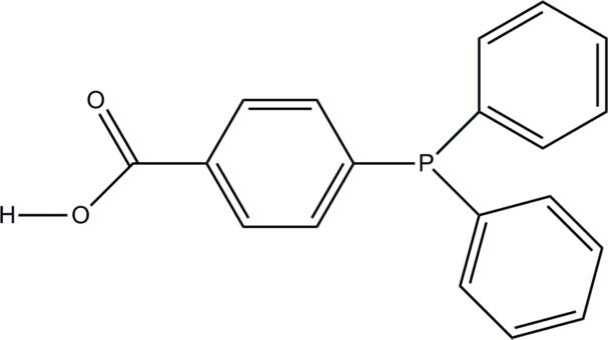


## Experimental

### 

#### Crystal data

C_19_H_15_O_2_P
*M* *_r_* = 306.28
Monoclinic, 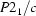

*a* = 7.885 (2) Å
*b* = 28.629 (8) Å
*c* = 7.066 (2) Å
β = 97.338 (4)°
*V* = 1581.8 (8) Å^3^
*Z* = 4
Mo *K*α radiation
μ = 0.18 mm^−1^
*T* = 113 K
0.24 × 0.20 × 0.20 mm

#### Data collection

Rigaku Saturn724 CCD diffractometer
Absorption correction: multi-scan (*CrystalClear*; Rigaku/MSC, 2005 ▶) *T*_min_ = 0.959, *T*_max_ = 0.965
15613 measured reflections
3714 independent reflections
3066 reflections with *I* > 2σ(*I*)
*R*_int_ = 0.049

#### Refinement

*R*[*F* ^2^ > 2σ(*F* ^2^)] = 0.040
*wR*(*F* ^2^) = 0.109
*S* = 1.03
3714 reflections
200 parameters
H-atom parameters constrained
Δρ_max_ = 0.25 e Å^−3^
Δρ_min_ = −0.30 e Å^−3^

### 

Data collection: *CrystalClear* (Rigaku/MSC, 2005 ▶); cell refinement: *CrystalClear*; data reduction: *CrystalClear*; program(s) used to solve structure: *SHELXS97* (Sheldrick, 2008 ▶); program(s) used to refine structure: *SHELXL97* (Sheldrick, 2008 ▶); molecular graphics: *SHELXTL* (Sheldrick, 2008 ▶); software used to prepare material for publication: *SHELXL97*.

## Supplementary Material

Crystal structure: contains datablock(s) global, I. DOI: 10.1107/S1600536811034234/hb6381sup1.cif

Structure factors: contains datablock(s) I. DOI: 10.1107/S1600536811034234/hb6381Isup4.hkl

Supplementary material file. DOI: 10.1107/S1600536811034234/hb6381Isup3.cml

Additional supplementary materials:  crystallographic information; 3D view; checkCIF report

## Figures and Tables

**Table 1:** Hydrogen-bond geometry (Å, °)

*D*—H⋯*A*	*D*—H	H⋯*A*	*D*⋯*A*	*D*—H⋯*A*
O1—H1⋯O2^i^	0.84	1.79	2.6190 (16)	170

Symmetry code: (i) 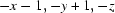.

**Fig. 1.**
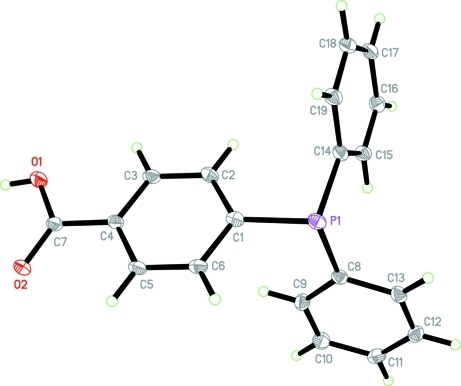
The molecular structure of (I). Displacement ellipsoids are drawn at the 30% probability level and H atoms are shown as small spheres of arbitrary radii.

**Fig. 2.**
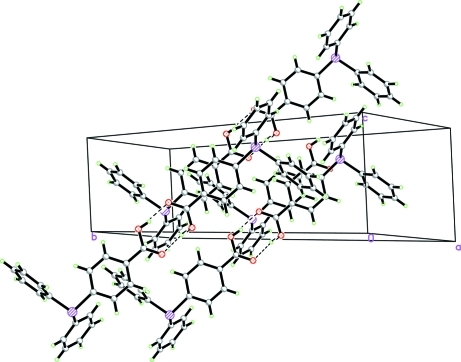
The crystal packing for (I). ,,;n,

## supplementary crystallographic information

### Comment

Phosphine ligands are important intermediates in organic chemistry e.g. (Dydio *et al.*, 2011), Water-soluble phosphines with the hydrophobic group are the most common phosphine lingands used in catalytic and biomedical aspects (Katti *et al.*, 1999; Pinault & Bruce, 2003). The title compound, (I), belongs to the fuctionalized water-soluble phosphines.

The O—H···O hydrogen bonds between the O atom of the carbonyl group and the H atom of the carboxyl group link the molecules into inversion dimers (Table 1).

### Experimental

4-iodobenzoic acid (5.0 mmol) and Et3N (10 mmol) were dissolved in CH3CN (30 ml). After the addition of Pb(OAc)2 (0.005 mmol) and Ph2PH (5.0 mmol), the reaction mixture was refluxed for 12 h. All volatiles were removed *in vacuo* and the obtained residue was dissolved in H2O (15 ml). After addition of KOH (10.0 mmol), the solution was extracted with Et2O. The aqueous solution was acidified with 2 N HCl and again extracted with Et2O. The collected ethereal phases were washed with H2O, dried over MgSO4 and evaporated to get a white precipite.  Colourless prisms of (I) were obtained by recrystallization from MeOH at room temperature.

### Refinement

All the H atoms were positioned geometrically (O—H = 0.84 Å, C—H = 0.95 Å) and refined as riding with *U*_iso_(H) = 1.2*U*_eq_(C) or 1.5*U*_eq_(O).

### Crystal data

**Table:**

C_19_H_15_O_2_P	*F*(000) = 640
*M**_r_* = 306.28	*D*_x_ = 1.286 Mg m^−^^3^
Monoclinic, *P*2_1_/*c*	Mo *K*α radiation, λ = 0.71073 Å
*a* = 7.885 (2) Å	Cell parameters from 5302 reflections
*b* = 28.629 (8) Å	θ = 1.4–28.0°
*c* = 7.066 (2) Å	µ = 0.18 mm^−^^1^
β = 97.338 (4)°	*T* = 113 K
*V* = 1581.8 (8) Å^3^	Prism, colorless
*Z* = 4	0.24 × 0.20 × 0.20 mm

### Data collection

**Table:**

Rigaku Saturn724 CCD diffractometer	3714 independent reflections
Radiation source: rotating anode	3066 reflections with *I* > 2σ(*I*)
multilayer	*R*_int_ = 0.049
Detector resolution: 14.22 pixels mm^-1^	θ_max_ = 27.9°, θ_min_ = 1.4°
ω and φ scans	*h* = −10→10
Absorption correction: multi-scan (*CrystalClear*; Rigaku/MSC, 2005)	*k* = −37→36
*T*_min_ = 0.959, *T*_max_ = 0.965	*l* = −9→8
15613 measured reflections	

### Refinement

**Table:**

Refinement on *F*^2^	Primary atom site location: structure-invariant direct methods
Least-squares matrix: full	Secondary atom site location: difference Fourier map
*R*[*F*^2^ > 2σ(*F*^2^)] = 0.040	Hydrogen site location: inferred from neighbouring sites
*wR*(*F*^2^) = 0.109	H-atom parameters constrained
*S* = 1.03	*w* = 1/[σ^2^(*F*_o_^2^) + (0.0573*P*)^2^ + 0.046*P*] where *P* = (*F*_o_^2^ + 2*F*_c_^2^)/3
3714 reflections	(Δ/σ)_max_ < 0.001
200 parameters	Δρ_max_ = 0.25 e Å^−^^3^
0 restraints	Δρ_min_ = −0.30 e Å^−^^3^

### Special details

**Table:**

Geometry. All e.s.d.'s (except the e.s.d. in the dihedral angle between two l.s. planes) are estimated using the full covariance matrix. The cell e.s.d.'s are taken into account individually in the estimation of e.s.d.'s in distances, angles and torsion angles; correlations between e.s.d.'s in cell parameters are only used when they are defined by crystal symmetry. An approximate (isotropic) treatment of cell e.s.d.'s is used for estimating e.s.d.'s involving l.s. planes.
Refinement. Refinement of *F*^2^ against ALL reflections. The weighted *R*-factor *wR* and goodness of fit *S* are based on *F*^2^, conventional *R*-factors *R* are based on *F*, with *F* set to zero for negative *F*^2^. The threshold expression of *F*^2^ > σ(*F*^2^) is used only for calculating *R*-factors(gt) *etc*. and is not relevant to the choice of reflections for refinement. *R*-factors based on *F*^2^ are statistically about twice as large as those based on *F*, and *R*- factors based on ALL data will be even larger.

### Fractional atomic coordinates and isotropic or equivalent isotropic displacement parameters (Å^2^)

**Table:**

	*x*	*y*	*z*	*U*_iso_*/*U*_eq_	
P1	0.20334 (5)	0.395759 (13)	0.81304 (6)	0.02428 (13)	
O1	−0.31468 (15)	0.46619 (4)	0.02047 (16)	0.0387 (3)	
H1	−0.3963	0.4778	−0.0527	0.058*	
O2	−0.45337 (13)	0.49967 (3)	0.24140 (15)	0.0326 (3)	
C1	0.04771 (17)	0.41954 (5)	0.6200 (2)	0.0232 (3)	
C2	0.04539 (18)	0.40779 (5)	0.4285 (2)	0.0262 (3)	
H2	0.1286	0.3867	0.3921	0.031*	
C3	−0.07624 (18)	0.42632 (5)	0.2905 (2)	0.0259 (3)	
H3	−0.0758	0.4181	0.1603	0.031*	
C4	−0.19939 (17)	0.45701 (5)	0.3423 (2)	0.0234 (3)	
C5	−0.19788 (18)	0.46940 (5)	0.5334 (2)	0.0245 (3)	
H5	−0.2817	0.4903	0.5696	0.029*	
C6	−0.07421 (18)	0.45129 (5)	0.6702 (2)	0.0248 (3)	
H6	−0.0719	0.4605	0.7997	0.030*	
C7	−0.33261 (18)	0.47604 (5)	0.1953 (2)	0.0261 (3)	
C8	0.07195 (17)	0.35227 (5)	0.9178 (2)	0.0221 (3)	
C9	−0.09792 (18)	0.34255 (5)	0.8492 (2)	0.0268 (3)	
H9	−0.1503	0.3581	0.7382	0.032*	
C10	−0.19171 (19)	0.31060 (5)	0.9399 (2)	0.0302 (4)	
H10	−0.3080	0.3048	0.8920	0.036*	
C11	−0.1167 (2)	0.28692 (5)	1.1006 (2)	0.0297 (3)	
H11	−0.1803	0.2646	1.1618	0.036*	
C12	0.0520 (2)	0.29624 (5)	1.1706 (2)	0.0295 (3)	
H12	0.1042	0.2802	1.2806	0.035*	
C13	0.14521 (18)	0.32874 (5)	1.0817 (2)	0.0266 (3)	
H13	0.2603	0.3351	1.1326	0.032*	
C14	0.33456 (17)	0.35885 (5)	0.6768 (2)	0.0233 (3)	
C15	0.31532 (17)	0.31046 (5)	0.6575 (2)	0.0250 (3)	
H15	0.2309	0.2949	0.7187	0.030*	
C16	0.41838 (19)	0.28490 (5)	0.5498 (2)	0.0271 (3)	
H16	0.4057	0.2520	0.5401	0.033*	
C17	0.53902 (18)	0.30724 (5)	0.4571 (2)	0.0281 (3)	
H17	0.6082	0.2897	0.3822	0.034*	
C18	0.55916 (18)	0.35529 (5)	0.4733 (2)	0.0286 (3)	
H18	0.6411	0.3708	0.4079	0.034*	
C19	0.45977 (17)	0.38077 (5)	0.5848 (2)	0.0264 (3)	
H19	0.4770	0.4135	0.5989	0.032*	

### Atomic displacement parameters (Å^2^)

**Table:**

	*U*^11^	*U*^22^	*U*^33^	*U*^12^	*U*^13^	*U*^23^
P1	0.0209 (2)	0.0242 (2)	0.0281 (2)	−0.00146 (13)	0.00446 (15)	−0.00451 (15)
O1	0.0387 (7)	0.0520 (7)	0.0271 (6)	0.0213 (5)	0.0104 (5)	0.0035 (5)
O2	0.0329 (6)	0.0343 (6)	0.0329 (6)	0.0142 (4)	0.0136 (5)	0.0035 (5)
C1	0.0228 (7)	0.0185 (6)	0.0295 (8)	−0.0019 (5)	0.0077 (6)	−0.0022 (6)
C2	0.0253 (7)	0.0236 (7)	0.0308 (8)	0.0051 (5)	0.0071 (6)	−0.0041 (6)
C3	0.0282 (7)	0.0253 (7)	0.0261 (8)	0.0035 (6)	0.0105 (6)	−0.0032 (6)
C4	0.0234 (7)	0.0201 (6)	0.0285 (8)	0.0018 (5)	0.0106 (6)	0.0026 (6)
C5	0.0249 (7)	0.0190 (6)	0.0323 (9)	0.0022 (5)	0.0143 (6)	0.0002 (6)
C6	0.0281 (7)	0.0224 (7)	0.0261 (8)	−0.0005 (5)	0.0119 (6)	−0.0023 (6)
C7	0.0275 (7)	0.0227 (7)	0.0305 (9)	0.0042 (5)	0.0126 (6)	0.0028 (6)
C8	0.0217 (7)	0.0232 (7)	0.0217 (8)	0.0015 (5)	0.0038 (5)	−0.0049 (6)
C9	0.0243 (7)	0.0301 (7)	0.0255 (8)	−0.0008 (6)	0.0008 (6)	0.0034 (6)
C10	0.0260 (8)	0.0323 (8)	0.0323 (9)	−0.0033 (6)	0.0037 (6)	0.0014 (7)
C11	0.0371 (9)	0.0264 (7)	0.0276 (9)	0.0009 (6)	0.0121 (7)	0.0003 (6)
C12	0.0381 (9)	0.0291 (8)	0.0214 (8)	0.0092 (6)	0.0042 (6)	0.0000 (6)
C13	0.0241 (7)	0.0302 (8)	0.0249 (8)	0.0061 (6)	0.0004 (6)	−0.0065 (6)
C14	0.0177 (6)	0.0264 (7)	0.0254 (8)	0.0013 (5)	0.0012 (5)	−0.0015 (6)
C15	0.0216 (7)	0.0257 (7)	0.0279 (8)	−0.0022 (5)	0.0039 (6)	0.0002 (6)
C16	0.0268 (7)	0.0248 (7)	0.0297 (9)	0.0028 (5)	0.0035 (6)	−0.0016 (6)
C17	0.0224 (7)	0.0345 (8)	0.0274 (8)	0.0064 (6)	0.0031 (6)	−0.0021 (6)
C18	0.0195 (7)	0.0338 (8)	0.0330 (9)	−0.0004 (5)	0.0060 (6)	0.0047 (7)
C19	0.0205 (7)	0.0261 (7)	0.0328 (9)	−0.0023 (5)	0.0037 (6)	0.0013 (6)

### Geometric parameters (Å, °)

**Table:**

P1—C8	1.8348 (15)	C9—H9	0.9500
P1—C14	1.8351 (15)	C10—C11	1.388 (2)
P1—C1	1.8443 (15)	C10—H10	0.9500
O1—C7	1.2920 (19)	C11—C12	1.384 (2)
O1—H1	0.8400	C11—H11	0.9500
O2—C7	1.2449 (17)	C12—C13	1.385 (2)
C1—C2	1.392 (2)	C12—H12	0.9500
C1—C6	1.4012 (19)	C13—H13	0.9500
C2—C3	1.383 (2)	C14—C19	1.3980 (19)
C2—H2	0.9500	C14—C15	1.398 (2)
C3—C4	1.3927 (19)	C15—C16	1.390 (2)
C3—H3	0.9500	C15—H15	0.9500
C4—C5	1.395 (2)	C16—C17	1.379 (2)
C4—C7	1.484 (2)	C16—H16	0.9500
C5—C6	1.383 (2)	C17—C18	1.388 (2)
C5—H5	0.9500	C17—H17	0.9500
C6—H6	0.9500	C18—C19	1.388 (2)
C8—C9	1.3930 (19)	C18—H18	0.9500
C8—C13	1.399 (2)	C19—H19	0.9500
C9—C10	1.384 (2)		
			
C8—P1—C14	101.89 (7)	C9—C10—C11	120.28 (14)
C8—P1—C1	101.07 (6)	C9—C10—H10	119.9
C14—P1—C1	101.02 (7)	C11—C10—H10	119.9
C7—O1—H1	109.5	C12—C11—C10	119.20 (14)
C2—C1—C6	118.58 (13)	C12—C11—H11	120.4
C2—C1—P1	123.67 (11)	C10—C11—H11	120.4
C6—C1—P1	117.75 (11)	C11—C12—C13	120.57 (14)
C3—C2—C1	120.97 (13)	C11—C12—H12	119.7
C3—C2—H2	119.5	C13—C12—H12	119.7
C1—C2—H2	119.5	C12—C13—C8	120.79 (13)
C2—C3—C4	120.02 (14)	C12—C13—H13	119.6
C2—C3—H3	120.0	C8—C13—H13	119.6
C4—C3—H3	120.0	C19—C14—C15	118.26 (13)
C3—C4—C5	119.67 (13)	C19—C14—P1	117.67 (11)
C3—C4—C7	120.17 (13)	C15—C14—P1	124.07 (11)
C5—C4—C7	120.15 (13)	C16—C15—C14	120.74 (14)
C6—C5—C4	119.94 (13)	C16—C15—H15	119.6
C6—C5—H5	120.0	C14—C15—H15	119.6
C4—C5—H5	120.0	C17—C16—C15	120.19 (14)
C5—C6—C1	120.78 (14)	C17—C16—H16	119.9
C5—C6—H6	119.6	C15—C16—H16	119.9
C1—C6—H6	119.6	C16—C17—C18	119.93 (14)
O2—C7—O1	123.34 (14)	C16—C17—H17	120.0
O2—C7—C4	120.86 (14)	C18—C17—H17	120.0
O1—C7—C4	115.80 (13)	C19—C18—C17	120.06 (14)
C9—C8—C13	117.94 (13)	C19—C18—H18	120.0
C9—C8—P1	124.24 (11)	C17—C18—H18	120.0
C13—C8—P1	117.76 (10)	C18—C19—C14	120.79 (14)
C10—C9—C8	121.20 (13)	C18—C19—H19	119.6
C10—C9—H9	119.4	C14—C19—H19	119.6
C8—C9—H9	119.4		
			
C8—P1—C1—C2	−102.98 (13)	C1—P1—C8—C13	−175.39 (11)
C14—P1—C1—C2	1.61 (13)	C13—C8—C9—C10	−0.1 (2)
C8—P1—C1—C6	77.23 (12)	P1—C8—C9—C10	−177.35 (11)
C14—P1—C1—C6	−178.18 (11)	C8—C9—C10—C11	−1.0 (2)
C6—C1—C2—C3	−1.1 (2)	C9—C10—C11—C12	1.1 (2)
P1—C1—C2—C3	179.08 (11)	C10—C11—C12—C13	−0.1 (2)
C1—C2—C3—C4	−0.4 (2)	C11—C12—C13—C8	−1.0 (2)
C2—C3—C4—C5	0.9 (2)	C9—C8—C13—C12	1.1 (2)
C2—C3—C4—C7	−178.49 (13)	P1—C8—C13—C12	178.52 (11)
C3—C4—C5—C6	0.1 (2)	C8—P1—C14—C19	−176.36 (10)
C7—C4—C5—C6	179.51 (12)	C1—P1—C14—C19	79.70 (11)
C4—C5—C6—C1	−1.7 (2)	C8—P1—C14—C15	4.54 (13)
C2—C1—C6—C5	2.2 (2)	C1—P1—C14—C15	−99.40 (12)
P1—C1—C6—C5	−178.05 (10)	C19—C14—C15—C16	0.10 (19)
C3—C4—C7—O2	172.69 (14)	P1—C14—C15—C16	179.19 (10)
C5—C4—C7—O2	−6.7 (2)	C14—C15—C16—C17	−1.4 (2)
C3—C4—C7—O1	−6.9 (2)	C15—C16—C17—C18	0.8 (2)
C5—C4—C7—O1	173.76 (13)	C16—C17—C18—C19	0.9 (2)
C14—P1—C8—C9	−102.00 (13)	C17—C18—C19—C14	−2.2 (2)
C1—P1—C8—C9	1.90 (14)	C15—C14—C19—C18	1.7 (2)
C14—P1—C8—C13	80.71 (12)	P1—C14—C19—C18	−177.48 (10)

### Hydrogen-bond geometry (Å, °)

**Table:**

*D*—H···*A*	*D*—H	H···*A*	*D*···*A*	*D*—H···*A*
O1—H1···O2^i^	0.84	1.79	2.6190 (16)	170

Symmetry codes: (i) −*x*−1, −*y*+1, −*z*.
